# Fluidic Considerations of Measuring Intracranial Pressure Using an Open External Ventricular Drain

**DOI:** 10.7759/cureus.15324

**Published:** 2021-05-29

**Authors:** Peter G Beidler, Alexander Novokhodko, Laura M Prolo, Samuel Browd, Barry R Lutz

**Affiliations:** 1 Bioengineering, University of Washington, Seattle, USA; 2 Mechanical Engineering, University of Washington, Seattle, USA; 3 Neurosurgery, Stanford University School of Medicine, Stanford, USA; 4 Surgical Services, VA Palo Alto Health Care System, Palo Alto, USA; 5 Neurosurgery, Seattle Children's Hospital, Seattle, USA; 6 Neurological Surgery, University of Washington, Seattle, USA

**Keywords:** external ventricular drain, cerebrospinal fluid, intracranial hypertension, continuous measurement, traumatic brain injury, catheter, bioengineering, neuro-surgery, neuro-monitoring, neurology and critical care

## Abstract

Measurement of intracranial pressure (ICP) during cerebrospinal fluid (CSF) drainage with an external ventricular drain (EVD) typically requires stopping the flow during measurement. However, there may be benefits to simultaneous ICP measurement and CSF drainage. Several studies have evaluated whether accurate ICP measurements can be obtained while the EVD is open. They report differing outcomes when it comes to error, and hypothesize several sources of error. This study presents an investigation into the fluidic sources of error for ICP measurement with concurrent drainage in an EVD.

Our experiments and analytical model both show that the error in pressure measurement increases linearly with flow rate and is not clinically significant, regardless of drip chamber height. At physiologically relevant flow rates (40 mL/hr) and ICP set points (13.6 - 31.3 cmH_2_O or 10 - 23 mmHg), our model predicts an underestimation of 0.767 cmH_2_O (0.56 mmHg) with no observed data point showing error greater than 1.09 cmH_2_O (0.8 mmHg) in our experiment. We extrapolate our model to predict a realistic worst-case clinical scenario where we expect to see a mean maximum error of 1.06 cmH_2_O (0.78 mmHg) arising from fluidic effects within the drainage system for the most resistive catheter.

Compared to other sources of error in current ICP monitoring, error in pressure measurement due to drainage flow is small and does not prohibit clinical use. However, other effects such as ventricular collapse or catheter obstruction could affect ICP measurement under continuous drainage and are not investigated in this study.

## Introduction

Cerebrospinal fluid (CSF) is a clear fluid found in the brain and spine which is produced by ependymal cells of the choroid plexus of the ventricles among other sites and is absorbed in the arachnoid granulations [1,2]. Approximately 500 mL of CSF is generated daily and either overproduction of CSF or decreased absorption can lead to elevated intracranial pressure (ICP), clinically known as hydrocephalus [2]. External ventricular drains (EVDs) are devices used to provide an alternative pathway of CSF egress from the ventricles and to monitor ICP. Treatment of high ICP after traumatic brain injury or due to other causes of hydrocephalus is the primary indication for the use of EVDs [3]. However, manufacturers list other indications. These include temporary drainage during infection, procedures to repair thoracic aortic aneurysm and thoraco-abdominal aortic aneurysm, monitoring ICP in surgical procedures, hemorrhage, and Reyes syndrome and related conditions [4]. CSF flows from the ventriculostomy drain through a distal catheter into a drip chamber. The drip chamber, which is maintained at atmospheric pressure, can be set at different heights to control CSF flow. CSF flows outward when the ICP is greater than the height difference multiplied by the mass density of fluid and the gravitational acceleration.

The typical method for making pressure measurements from an EVD is done by turning a stopcock at the T-connector to stop fluid flow and connect the catheter to an attached pressure transducer [5]. If drainage is needed, a healthcare worker manually closes the stopcock for each measurement and reopen it afterwards [6]. In many cases, this leads to a tradeoff between frequency of pressure measurements, drainage, and healthcare resources. Often a patient needs to be maintained in the intensive care unit if pressure measurements are desired in addition to CSF drainage.

Continuous pressure measurements with concurrent drainage are possible by turning the stopcock so that fluid can pass through to the drip chamber and simultaneously have connection to the pressure transducer. Simultaneous drainage and ICP measurement has not been adopted clinically given concern for sources of error in these pressure measurements, however, the error has not been well-characterized. Proposed benefits of continuous monitoring include monitoring cerebrovascular autoregulation by pressure reactivity index without the need for an additional probe and rapid detection of CSF flow blockages and other emergencies that may place the patient at risk [6,7].

The sources of error that affect EVD pressure measurements as they are currently made include pressure heterogeneity throughout the brain, height error in EVD transducer placement, collapsed ventricles obstructing the catheter fenestrations, and debris or bubbles in the drainage line. It has been shown that pressures can vary by as much as 8.2 cmH_2_O (6 mmHg) in different parts of the brain [8]. In order for pressure measurements to be accurate, the pressure transducer, which is attached at the zero point on the EVD must be placed accurately at the height of the external auditory meatus (EAM). Bisnaire et al. showed that placement height error can be as great as 4.4 cm on average and is highly dependent on nurse experience and which tools they use for leveling (e.g., eyeball, string, laser level) [9]. This height error corresponds to a pressure error of 4.40 cmH_2_O (3.26 mmHg) assuming a CSF density of 1.00059 g/mL [10]. Air bubbles can cause large, unpredictable errors [11]. Lastly, obstructions in the drainage line can cause drastic underestimations in pressure depending on the size of occlusion [12]. Thus, there are multiple sources of error that can each contribute several cmH_2_O error in ICP measurement.

Measurements made through EVDs with concurrent flow are subject to additional errors caused by dynamic fluid effects and ventricular collapse [13]. The dominant fluidic effect is fluidic resistance to flow through the catheter, which would cause a decrease in pressure at the transducer as a result of flow. Additionally, it has been predicted that errors resulting from partial obstruction of catheter fenestrations will cause greater pressure underestimation when measuring with concurrent flow [14]. Ventricular collapse may cause this.

This study investigates the impact of fluidic effects on the accuracy of pressure measurements with drainage. It is important to note that additional factors in clinical practice may affect error in measurement, but our goal is to clarify the baseline contributions to error from an engineering perspective since they have not been clearly described.

## Materials and methods

Head model

**Figure 1:**
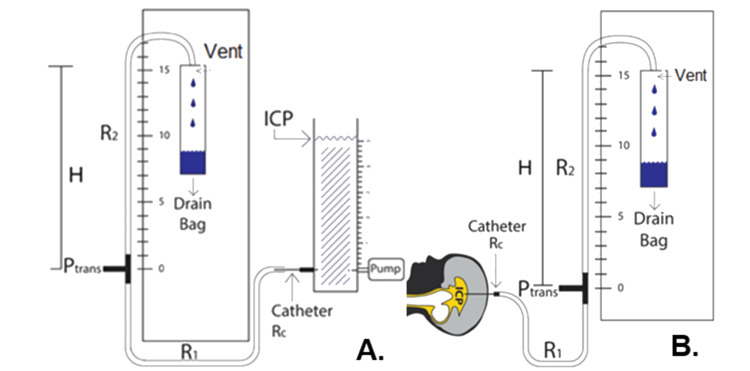
Comparison of Experimental and Clinical Setup A: Experimental setup for EVD characterization. The head model was fed by a syringe pump with programmable flow rate and fluid drained from the head model directly into a catheter which was placed into the column as shown. This junction was set at the same height as the pressure transducer. P_trans_ corresponds to the pressure measured with a digital transducer at the same height as the zero point. R_1_ and R_2_ are the fluidic resistances of tubing segments before and after the transducer. R_c_ is the resistance of the Medtronic 9025 catheter (Medtronic, Minneapolis, USA). Since the drip chamber is vented, pressure in it is equal to atmospheric pressure (zero gauge pressure). H is the distance above the zero point that the tubing terminates. B: Diagram of standard EVD setup. The zero point on the scale is set to the same height as the patient’s external auditory meatus by medical staff and the patient head is immobilized. EVD: external ventricular drain; ICP: intracranial pressure

The head model (Figure 1A) consisted of an acrylic tube that was fed by an automated syringe pump at its base. A clinical EVD setup is shown in Figure 1B for comparison. A ventricular catheter was inserted into the acrylic tube at the same height as the syringe pump. The height of water above the catheter attachment corresponded to ICP.

Electronics

The electronic components used in this study are listed in Table 1. The pressure transducer was manually calibrated by measuring the voltage at given pressures. Data collection and voltage supply to the pressure transducer was done via a National Instruments DAQ (data acquisition system) (National Instruments, Austin, USA), and all data was processed in LabVIEW 2018 (National Instruments) and MATLAB R2018a (MathWorks, Inc., Natick, USA).

**Table 1:** Electronic components DAQ: data acquisition system

Component	Name	Functional Accuracy
Transducer	PX409-001G5V (Omega Engineering, Inc., Norwalk, USA)	± 0.0557 cmH2O (0.041 mmHg)
DAQ	NI USB-6259 (National Instruments)	± 4.5 μV
Syringe pump	NE-300 (New Era Pump Systems, Inc., Farmingdale, USA)	± 1%

CSF simulant

For all experiments, water was used to simulate CSF. CSF has been reported to have a similar viscosity to water, within 1-8% depending on protein concentration [15]. Another source found CSF viscosity to be between 0.7-1 mPa*s [16]. For comparison, the viscosity of water is 0.70 mPa*s at 37 °C [15]. Our tests were done at room temperature, where the viscosity of water is 0.93 mPa*s [17]. Both values are within the normal CSF range. Also, like water, CSF behaves as a Newtonian fluid [16,18], meaning that it does not change its viscosity when moving [17].

Testing parameters

The pressure and flow rate parameters used in this study are based on values reported in the literature, shown in Table 2.

**Table 2:** Test parameter literature search EVD: external ventricular drain; ICP: intracranial pressure; CSF: cerebrospinal fluid

Parameter	Magnitude
EVD-relevant ICP range	Up to 30 mmHg [13]
Hypertension threshold	20 mmHg [19]
	20-30 mmHg [20]
	22 mmHg [3]
CSF production rate	18-24 mL/hr [21]
	22 mL/hr [22]
	18-36 mL/hr [23]
	12-30 mL/hr [13]

Catheter

The Medtronic 9025 catheter was selected for this study because it is at the upper end of estimated resistances of most ventricular catheters in clinical use [24]. Five commonly used catheters’ dimensions are listed in Table 3.

**Table 3:** Dimensions and Resistances of Common Catheters * Integra LifeSciences Corporation, Princeton, USA

Catheter	Length	Inner diameter	Length of fenestrated region	Estimated Resistance (Pa*s/m^3^)
Medtronic 9025	23 cm	1.3 mm	12 mm	3.26E+09
Codman 82-1221*	15 cm	1.3 mm	10 mm	2.13E+09
Medtronic 27251	23 cm	1.2 mm	8 mm	4.48E+09
Medtronic 41715	15 cm	1.5 mm	10 mm	1.20E+09
Medtronic 27771	20 cm	1.3 mm	24 mm	2.83E+09

Catheter resistance measurements

Resistance measurements were made for segments of tubing in the Medtronic Duet External Drainage and Monitoring System as well as the Medtronic catheter model 9025 (**R_c_**). The EVD was divided into segments of tubing (Figure 1A) between the catheter and transducer (**R_1_**) and between the transducer and drip chamber (**R_2_**). To make these measurements, a flow rate (**Q**) of 360 mL/hr was pumped into the head model and fluid drained through the tubing of interest into atmospheric pressure with zero height difference. The height of water in the head model (ICP) was allowed to reach steady state. The high flow rate relative to physiologic CSF production was chosen to increase observed pressures, increasing accuracy of our measurements. At this flow rate, a maximum Reynold’s number of 1.8*10^-3^ was calculated, meaning flow remained laminar so resistance values would not be affected by the high flow rate. All experiments were done in triplicate. Resistance was calculated by R = P/Q, where P is the pressure difference across the tubing segment. This is analogous to Ohm’s law and can be derived from the Hagen-Poiseuille equation [17].

Simulation of open drain pressure measurement

The error in pressure measurements with concurrent drainage was evaluated experimentally using an open-loop setup shown in Figure 1A. This EVD was set up in the conventional manner, and then the stopcock was switched to allow drainage and pressure measurement simultaneously. The head model was fed by a syringe pump and drained into the EVD through the Medtronic 9025 catheter. A digital transducer (PX409) was attached at the T-connector, which was fixed at the zero height point. All bubbles were purged from the system. Inflow rates spanning the range from 0-240 mL/hour were tested. Although this range is much greater than physiological CSF production rates, higher rates were included in the study to help validate the linear model and to test the effects of high CSF flow rates that may occur transiently. For each flow rate, the drip chamber height (**H**) was set to create clinically relevant ICP values of 10, 19, and 23 mmHg. This corresponds to 13.6, 25.8, and 31.3 cmH_2_O. That is also the value of *H* in cm above the head model. For each pressure/flow rate combination, the flow rate was set and ICP was allowed to reach a steady-state. Steady-state was considered less than 0.1 cmH_2_O (0.074 mmHg) change in seven minutes. The steady-state ICP and pressure at the T-connector were recorded. These experiments were done in triplicate, and to ensure that steady-state was reached, the second trial of each set was done in reverse order from the first and third. This means that the height of water in the column increased to equilibrium in the first and third trials and decreased to equilibrium in the second trial. If steady-state was not reached, observed pressure errors would be systemically lower in the second trial than in first and third, and the observed error would be higher overall than the true error.

Model development

Theoretical Catheter Resistances

Estimated catheter resistances were calculated by the Hagen-Poiseuille equation, using the dynamic viscosity of water at 25ºC (0.93 mPa*s) [17].The catheters were modeled as cylindrical tubes as long as the entire catheter, thus ignoring the fenestrations. While this should be fairly representative of measured resistances, this estimate does not account for a few factors. First, it is unclear without in-depth computational fluid dynamics simulations, how fenestrations affect catheter resistance. It has been shown computationally that most of the fluid travels through the most proximal holes of catheters [24], meaning there is less tubing to create fluid resistance, but it is difficult to approximate how much resistance the fenestrations themselves add. EVD tubing segments were also modeled as cylindrical tubes and fittings were ignored because all fittings had equal or greater inner diameters than the segments of tubing they joined and were comparatively very short. This included T-connectors and a larger tube into which the catheter tip was placed. This analysis ignores the potential effects of tubing coiling on the cross-sectional geometry as well as potential flow separation caused by rough transitions between fitting and tubing. While these errors are expected to be small, we also measured fluid resistance of each component experimentally, as described above, to confirm our theoretical estimates.

Prediction of Measurement Error

To better understand the results of our experiment, we also performed a hydrodynamic analysis of the system to predict error in pressure measurements. As before, the system was examined in three segments, **R_c_**, **R_1_**, and **R_2_**. Equation 1 is derived through a simple pressure analysis of the EVD system. In summary, it states that ICP is greater than atmospheric pressure because of fluidic resistance (**QR_total_**) and fluid head (**ρgH**) created by the raised drip chamber. Applying the same analysis to only the proximal section, we can isolate the difference between ICP and pressure at the transducer, which is given by Equation 2**.** In Equation 1, **ρ** is the density of water (998.2 kg/m^3^), **g** represents gravitational acceleration at sea level (9.81 m/s^2^). **Q** is the flow rate of water through the drainage system, which is set by the syringe pump as long as the system is at steady state, **R_1_** is the resistance of the tubing portion between the head model and transducer in units of Pa*s/m^3^.

$$ICP = Q(R_1 + R_c) + QR_2 -\rho gh_2 (Eq. 1)$$

$$ICP-P_{trans} = Q(R_1+R_c) (Eq. 2)$$

This model makes a few important assumptions. First, we assume the system has reached a steady-state such that the flow rates in and out of the head model are equal, and therefore ICP is constant. This is maintained by the experimental procedure. Second, the pressure inside the EVD drip chamber is assumed to equal atmospheric pressure. This is maintained by a filtered air vent in the top of the drip chamber [25]. All pressures are gauge pressures reported relative to atmospheric pressure. Third, we assume all flow in the system is laminar. Lastly, we assume water behaves as a Newtonian, incompressible fluid. This is a commonly used approximation.

## Results

**Measured resistance values are comparable to expectations**. Table 4 shows dimensions for the components of our system, theoretical resistances modeled as described above and measured resistances calculated using the Hagen-Poiseuille equation. The measured resistances follow our simplified estimations. Measured resistance values are used to predict the error in pressure measurement.

**Table 4:** Comparison of Predicted and Measured Resistance Values for the Catheter and EVD Tubing EVD: external ventricular drain

Component	Inner Diameter (mm)	Length (m)	Predicted Resistance (Pa*s/m^3^)	Measured Resistance (Pa*s/m^3^)
Medtronic catheter 9025	1.3	0.23	2.9E+09	4.1E+09
EVD Tubing R_1_	1.8	1.2	4.1E+09	3.8E+09
EVD Tubing R_2_	1.8	0.5	2.9E+09	2.0E+09

**Our model predicts that error in pressure measurements will increase linearly with flow rate**. Measurements were made by the transducer in a traditional EVD. Equation 2 models this system, using the measured resistance values in Table 4 and the flow rates of interest. In the physiological range of flow rates, the greatest measurement error predicted for the Medtronic 9025 catheter is 0.891 cmH_2_O (0.655 mmHg), as seen in Table 4.

**Figure 2:**
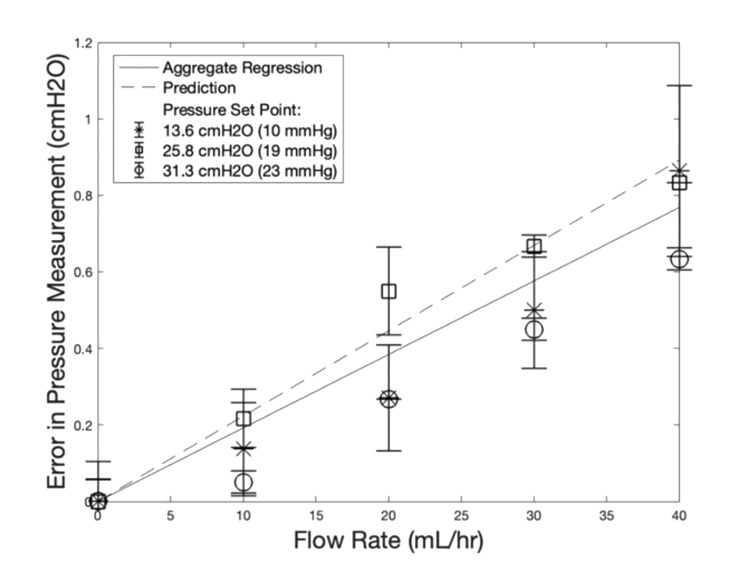
Error in Pressure Measurement Is Linear and Small Linear regressions of pressure measurement error compared to the prediction in a physiologic flow rate range. The analytical prediction follows experimental data, confirming that error in pressure measurements scales linearly with flow rate and is not affected by drip chamber height. Most importantly, the observed and predicted errors are very small, mostly under 1 cmH_2_O. Data is shown as mean ± standard deviation (n = 3). Slope values, statistical analysis and plots of results from higher flow rates and individual data points are included in Table 5.

**Experimental measurements agree with the linear relationship between error in pressure measurement and flow rate**. This can be seen in Figure 2. The true pressure is the height of fluid in the head model at steady state. The error is the difference between that physically determined quantity and the reading on the transducer. The linearity of all aggregate data, regardless of drip chamber height, is demonstrated by a Pearson correlation coefficient of 0.993. This correlation coefficient has a p-value of 0.00001. The slopes of the regression lines at each drip chamber height vary by 10.5% or less from each other. From the data in Table 5 and Figure 2, the predicted linear relationship can be seen.

**Table 5:** Analysis of Pressure and Flow Data ICP: intracranial pressure

Regressions table	13.6 cmH_2_O (10 mmHg)	25.8 cmH_2_O (19 mmHg)	31.3 cmH_2_O (23 mmHg)	Aggregate	Prediction
Slope (cmH_2_O/ (mL/hr))	0.0206	0.0187	0.0185	0.0192	0.0223
Standard error of slope (cmH_2_O/ (mL/hr))	0.000319	0.000286	0.000231	0.000185	
R^2^	0.994	0.994	0.996	0.993	
p-value of correlation	<0.000001	<0.000001	<0.000001	<0.000001	
ICP measurement error at 40 mL/hr	0.813 cmH_2_O (0.598 mmHg)	0.747 cmH_2_O (0.549 mmHg)	0.740 cmH_2_O (0.544 mmHg)	0.767 cmH_2_O (0.564 mmHg)	0.891 cmH_2_O (0.655 mmHg)

**The magnitude of error in pressure measurement with concurrent flow is small**. As described above, error in pressure measurement corresponds to the difference between the true pressure in the head model and the pressure reported by the transducer with the stopcock set to allow flow. In Figure 2, average errors are generally less than 1 cmH_2_O in magnitude at all drip chamber heights. Results slightly exceeding 1 cmH_2_O are only reported for 40 mL/hr.

**Figure 3:**
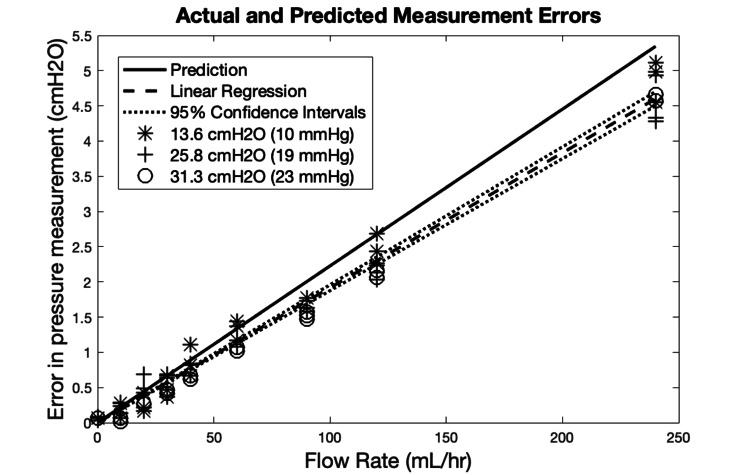
Error in Pressure Measurement Over a Wider Range of Flow Rates This linear regression of all aggregate data points across all drip chamber heights and flow rates has a slope of 0.0192 cmH_2_O*hr/mL and an R^2^ of 0.987. The regression has been constrained to have no offset. The Pearson correlation coefficient is r(81) = 0.9934, p = 0.00001. 95% confidence intervals are plotted alongside the analytical prediction. The prediction line is based on Equation 2 and has a slope of 0.0223 cmH_2_O*hr/mL.

**The aggregate data show a high degree of linearity, even at unphysiological flow rates**. This suggests that sources of nonlinearity such as turbulence would not come into play even during transient high flow episodes. It is notable that the greatest observed error within the physiologically relevant range of 0-40 mL/hr was 1.107 cmH_2_O (0.814 mmHg) as seen in Figure 3. The true error in pressure measurement is lower than the predicted error. From this data, we conclude that the error is not clinically significant in the physiological range for any tested drip chamber height.

## Discussion

Continuous EVD management offers significant advantage in the clinical setting and opens up the option to more easily monitor ICP in a non-ICU setting. Real time monitoring permits a rapid response to emergencies. Pulsation of blood vessels in the head can be detected in EVD pressure transducers in the absence of obstruction within the drainage line. A clinician could respond rapidly to an interruption in this signal. Perhaps even more impactful is the potential for completely automated drainage systems that monitor pressure and control the rate of drainage [26,27]. As the development of such systems is undoubtably on the horizon, it will become increasingly important to understand the sources of error in pressure monitoring with continuous drainage to characterize the efficacy of these devices and improve them. Several studies have investigated the accuracy of these measurements with clinical and benchtop experiments, but little is known about the contribution of different sources of error.

One clinical study involving 50 patients being treated with EVD investigated the accuracy of pressure measurements made from the drainage line with concurrent flow and compared them to the standard of care: Traditional fluid-coupled measurements made by stopping flow [6]. These authors found that the error due to flow was on average 2.2 cmH_2_O (1.6 mmHg) and was less than 4 cmH_2_O (3 mmHg) 97% of the time, concluding that it was negligible. However, these results may not be completely representative of ICP measurement accuracy with concurrent flow. The clinical scenario is complex and there are sources of error such as placement error or ventricular collapse that may affect traditional fluid-coupled measurements. An advantage of the simpler benchtop head model is that error due to flow can be studied in isolation, so other error sources do not confound the estimate.

A second clinical study of 20 patients simultaneously compared ICP measurements made through an EVD with concurrent flow against a dedicated air-pouch transducer placed into the ventricle [7]. Air-pouch transducers are not subject to many of the sources of error of fluid-coupled measurements and are considered more robust [12]. The authors reported that the difference between pressure measurements of the air-pouch and EVD transducers was not significant by T-test (p > 0.05). While the two measurements did not differ by T-test, in some cases the EVD transducer underestimated ICP in clinically significant way. Clinical intracranial hypertension is often defined as >20 mmHg [19], and the authors report that the EVD transducer failed to detect this in several instances. The conclusion of this study was that pressure measurement via EVD with concurrent flow can be useful but requires close surveillance for artifacts. See Table 2 for various definitions of intracranial hypertension given in the literature.

In addition to the clinical studies, a third study was performed using a physical model of the head and brain to assess EVD pressure measurement with concurrent flow [13]. This study concluded that making pressure measurements without stopping flow was unsafe because the EVD transducer would generally underestimate ICP, suggesting that it may fail to detect intracranial hypertension clinically. The difference in pressure between measured pressure and pressure in the brain was said to be a result of ventricular collapse and tubing resistance to flow. Tubing resistance due to flow, however, was not measured nor estimated. Additionally, the manuscript did not provide parameters of the head model representing properties of the brain and ventricle.

These three studies have mixed conclusions about the reliability of pressure measurement via EVD with concurrent flow and report the error of these measurements but do not characterize and quantify sources of error. The objective of this manuscript is to understand how fluidic effects contribute to the error during continuous pressure monitoring in an EVD with concurrent drainage. We investigated the error due to fluid flow. We found it to be small in comparison with other sources of error that affect measurements even when drainage is stopped. We showed that the error follows a predicted linear pattern, growing with flow rate.

Sources of error can be estimated as follows. First, the PX409 transducer is accurate to 0.056 cmH_2_O (0.041 mmHg). Additionally, pressure in the head model was measured by eye using graduations and is also accurate to 0.05 cmH_2_O (0.036 mmHg). Error in ICP measurement at the transducer is given by the difference between these two pressures. These sources of error are not correlated so there is no covariance between them. Thus, the errors sum to a total measurement error of 0.11 cmH_2_O (0.077 mmHg) . Additionally, as stated above, steady state was considered to be no movement greater than 0.1 cmH_2_O (0.074 mmHg) in seven minutes. Because measurements were made by eye, they were made every seven to 10 minutes, so it is possible that not all trials equilibrated for the same amount of time.

It should be noted that these experiments were not performed with CSF, nor the most resistive ventricular catheter on the market. Our findings can be extrapolated to predict the greatest possible error due to fluidic effects. CSF has been described as having a viscosity between 0.7-1 mPa*s [16] or being 1-8% more viscous than water [15] and one of the most resistive catheters, Medtronic 27251, is slightly narrower than the Medtronic 9025 catheter used in this study [24]. We expect the Medtronic 27251 to have a resistance around 4.48E+09 Pa*s/m^3^, compared with our experimentally determined resistance of 4.053 Pa*s/m^3^ for the Medtronic 9025. We model a worst-case CSF viscosity of 1.0 mPa*s. Using the analytical model validated in this study, we predict the error in the theoretical most erroneous case to be 1.06 cmH_2_O (0.78 mmHg).

To put this in context, Bisnaire et al. suggested an acceptable error of 2 cm for drip chamber height, which is used clinically to regulate ICP [9] and corresponds to a pressure error of 2 cmH_2_O (1.5 mmHg). Also, a literature review by Zacchetti et al. found a mean error of ~2.1 cmH_2_O (1.54 mmHg) in ICP between different locations in the brain, with 30% of readings exceeding 6 cmH_2_O (4.4 mmHg) of error between locations [8]. Another recent review suggested a necessary accuracy of ± 2.7 cmH_2_O (2 mmHg) for ICP monitoring techniques [28]. In comparison to these existing errors inherent to ICP measurements, the pressure measurement error found in our engineering analysis is relatively small.

While we have shown that the flow of fluid creates a small pressure head in the drainage line which equates to small errors in measurement, it should be emphasized that the flow of fluid could have other effects on pressure measurement. Other errors, such as ventricular collapse, were not investigated in this study and could have implications on the viability of clinical ICP measurements with concurrent flow. Because ventricular collapse, like tubing obstruction, is known to decouple pressure in the drainage line from that of the brain, these events could cause measurement artifacts. In the case of ventricular collapse, the suction of surrounding tissue against catheter fenestrations would create negative pressure in the drainage line causing measurements to underestimate ICP. Full or partial occlusions would likely have a similar effect due to an increase in the resistance R_c_ or R_1_. While these phenomena may cause significant error in pressure measurements, they could also affect traditional fluid-coupled measurements to some degree.

## Conclusions

The experimental data in this study confirm that the error in pressure measurements due to fluidic effects in ventricular drainage lines with concurrent flow follow a simple linear relationship as predicted by an analysis of laminar fluid mechanics. The error in pressure measurement is primarily due to the catheter and portion of tubing between the catheter and pressure transducer. For all measured drip chamber heights this error does not exceed commonly accepted clinical thresholds. Because of the small magnitude of this error demonstrated by both analysis and prediction, we conclude that fluidics are not an inherent barrier to pressure measurements in EVD without stopping flow. This has implications on current practice as well as the development of automated EVD systems. We must still acknowledge that other sources of error not investigated in this study, such as ventricular collapse and drainage line obstruction, must be further characterized in order to evaluate the validity of these pressure measurements for clinical implementation.
